# Identification of soybean mutants with low cesium accumulation, characterization of the causative gene, and field evaluation

**DOI:** 10.1270/jsbbs.24069

**Published:** 2025-08-09

**Authors:** Kyoko Takagi, Yuko Yokota, Yukiko Fujisawa, Susumu Hiraga, Hisaya Matsunami, Katashi Kubo, Akito Kaga, Toyoaki Anai, Masao Ishimoto

**Affiliations:** 1 Tohoku Agricultural Research Center, National Agriculture and Food Research Organization (NARO), 50 Harajukuminami, Arai, Fukushima, Fukushima 960-2156, Japan; 2 Institute of Crop Science, NARO, 2-1-2 Kannondai, Tsukuba, Ibaraki 305-8518, Japan; 4 Saga University, 1 Honjo-machi, Saga, Saga 840-8502, Japan

**Keywords:** soybean, cesium, induced mutant, salt overly sensitive (SOS) pathway

## Abstract

The radiocesium contamination of food poses a serious threat to food safety because radiocesium has a long half-life and emits harmful radiation during decay. Therefore, it is important to reduce radiocesium uptake by crops. In this study, we identified low-cesium-accumulating soybean mutants from an ethyl methanesulfonate-induced mutant population. Through the phenotypic screening of the population based on the seed cesium concentration, 10 candidate low-cesium-accumulating mutant lines were selected. Five of these exhibited significantly reduced seed radiocesium concentrations compared with the original variety, and one had accumulated an excessive amount of sodium. Since plant intracellular sodium ion homeostasis is regulated by the salt overly sensitive (SOS) pathway, sequence analysis of *GmSOS1*, which encodes an Na^+^/H^+^ antiporter in this pathway, revealed that the high-sodium-accumulating line contains a mutation in this gene. Additionally, two more *gmsos1* mutant lines were isolated from the mutant population. In the field trial, the three independent *gmsos1* mutant lines possessed lower seed cesium concentrations than the wild type. These results indicate that *GmSOS1* is responsible for seed cesium accumulation, and *gmsos1* mutants are potential breeding materials for reducing hazardous radiocesium accumulation in soybeans.

## Introduction

An enormous amount of radiocesium was released into the agricultural land in eastern Japan following the nuclear accident at the Tokyo Electric Power Company’s Fukushima Daiichi Nuclear Power Plant (TEPCO–FDNPP) in March 2011. Since radiocesium has a relatively long half-life (2.06 years for ^134^Cs and 30.2 years for ^137^Cs) and emits harmful β and γ radiation during its decay, strategies aimed at reducing radiocesium accumulation in crops should be developed and implemented. As the chemical properties of cesium (Cs) are similar to those of potassium (K), the uptake and distribution of Cs are considered to occur through the same transport mechanism used by K. In fact, increasing K fertilizer application has resulted in decreased radiocesium uptake by various crops such as rice and soybeans (Hirayama *et al.* 2018, Ishikawa *et al.* 2018, Kato *et al.* 2015, Matsunami *et al.* 2021). As continuous K fertilizer application is labor intensive and expensive, “safer” crops that accumulate a small amount of radiocesium should be cultivated to reduce radiocesium contamination of agricultural products.

Previously, the genetic variation for the seed Cs concentration was surveyed in soybeans (*Glycine max* L.) using a set of mini-core collections representing a large genetic diversity (Kaga *et al.* 2012) to enable the selection of candidate accessions with traits for low Cs accumulation (Takagi *et al.* 2015). Subsequently, Uda *et al.* (2021) selected ‘Onihadaka’ as a low-Cs-accumulating candidate from the same mini-core collection. Using quantitative trait loci-sequence analysis, these authors highlighted the candidate genes responsible for the difference in Cs uptake between ‘Enrei’ and ‘Onihadaka’. However, identifying the responsible gene is challenging because the two accessions are genetically diverse, and genes other than the target gene can also influence evaluation of the seed Cs concentration.

Induced mutations are useful for examining gene function and improving crops; therefore, several mutant populations have been developed in a range of crops, including rice, maize, wheat, barley, and soybean (Sikora *et al.* 2011). Since a mutant population is composed of lines with the same genetic background, it is easier to identify the effects of mutant genes and exploit them as breeding materials, rather than relying on genetically diversified genetic resources. Genetic variability, as a result of mutations induced by various mutagens, has contributed to modern plant breeding and has played a major role in the development of superior plant varieties with improved productivity and quality (Oladosu *et al.* 2016). For example, the ‘Kosuzu’ soybean cultivar, is a γ-ray induced direct-use mutant cultivar, which was selected due to its earlier maturity and improved lodging resistance against cv. ‘Natto Shouryu’, which possesses small seeds and is well suited for use in the “natto” fermentation process (Hashimoto *et al.* 1988). Induced mutants can therefore be used to improve superior varieties within a short period of time, rendering them useful in situations where a rapid response is required, such as in the development of “safer” crops. To date, various mutant soybean populations have been developed using chemical mutagens such as ethyl methanesulfonate (EMS) and N-nitroso-N-methylurea (NMU) (Anai 2012, Cooper *et al.* 2008, Li *et al.* 2017, Tsuda *et al.* 2015). For example, Tsuda *et al.* (2015) constructed a high-density mutant library consisting of the DNA and seeds of 1,536 plants by repeating the EMS treatment for a Japanese soybean cultivar ‘Enrei’. This library has been successfully applied to select mutants with a phenotype of interest based on phenotypic screening. In the case of saponin, a major secondary metabolite in soybean seeds, novel compositional variants have been obtained, such as the deletion of 2,3-dihydro-2,5-dihydroxy-6-methyl-4*H*-pyran-4-one (DDMP) saponin through phenotypic screening (Krishnamurthy *et al.* 2019).

Despite these advances, to the best of our knowledge, no mutant soybean lines with low Cs accumulation have been reported. However, several mutant lines with reduced Cs accumulation have been identified by the phenotypic screening of rice. For example, Ishikawa *et al.* (2017) demonstrated that a mutant of *OsSOS2*, which encodes a serine/threonine-protein kinase required for the salt overly sensitive (SOS) pathway, exhibited reduced radiocesium concentrations in its grains. Notably, the SOS signaling pathway, which comprises *SOS3*, *SOS2*, and *SOS1*, mediates cellular signaling under salt stress to maintain ion homeostasis. More specifically, *SOS3* encodes a myristoylated calcium-binding protein, wherein SOS3 interacts with and activates the serine/protein kinase SOS2, leading to activation of the downstream target SOS1, an Na^+^/H^+^ antiporter (Ji *et al.* 2013). The reduced Cs^+^ uptake in the *ossos2* mutant may be closely related to the lower expression levels of several K^+^ and Na^+^ transporter genes due to the K^+^/Na^+^ imbalance in the roots caused by the *OsSOS2* mutation (Ishikawa *et al.* 2017). The *ossos2* mutant exhibited no difference in yield compared to the wild type, indicating that phenotypic selection is a viable approach for obtaining mutants suitable for use as breeding materials.

With these considerations in mind, the current study aimed to identify mutant lines with low levels of Cs accumulation. To achieve this, the high-density mutant library was searched to identify mutant lines with low Cs accumulation levels, and five lines with consistently low Cs contents were identified. One of the selected lines was a mutant of *GmSOS1*, a homolog of *AtSOS1* and *OsSOS1*, which encode the Na^+^/H^+^ antiporter in the salt overly sensitive (SOS) pathway. A reduction in radiocesium levels in the field was subsequently confirmed using segregating populations derived from crosses between the original variety and *gmsos1* mutants. The findings of this study are expected to contribute to the breeding of soybean varieties with low Cs accumulation, to reduce radiocesium contamination in soybeans and soybean-derived. Furthermore, the successful identification of multiple low cesium lines through the phenotypic screening of the mutant population in this study suggests that this strategy can be applied not only to harmful cesium accumulation, but also to the improvement of important agricultural traits, such as disease resistance and seed composition in soybean.

## Materials and Methods

### Plant materials and field conditions

The EMS-induced mutant library of the Japanese cultivar ‘Enrei’ (Tsuda *et al.* 2015) was referred to in this study. This mutant library consisted of associated DNA and seeds from 1,536 M2ʹ plants. In the phenotypic screening, 905 lines were used, which provided >50 M3ʹ seeds.

In addition to the 10 lines selected through phenotypic screening, three *gmsos1* mutant lines (EnT-2953, EnT-3153, and EnT-3612) and their segregating populations (F_2_, F_3_, and F_4_) developed from the crosses between the *gmsos1* lines and the original variety (cv. Enrei) were used in field cultivation experiments. For this purpose, two fields in the Fukushima Prefecture with different soil characteristics were employed, namely Fields A and B (Table 1). Field A is a converted paddy field located 55 km northwest of TEPCO–FDNPP. The planting distance in Field A was 75 cm between rows and 15 cm between plants. Field B is an upland field located 65 km northwest of TEPCO–FDNPP. The planting distance in Field B was 75 cm between rows and 15 cm between plants.

### Analysis of ^133^Cs and other elements in the plant samples

In the phenotypic screening of low-Cs-accumulating lines from the mutant population, the half-cotyledon of eight seeds from each M3ʹ line was freeze-dried. The freeze-dried cotyledons were individually ground into fine powder using a multibead shocker (Yasui Kikai, Osaka, Japan). Each ground sample (20 mg) was placed in a 15 mL metal-free centrifuge tube (INA·OPTIKA, Osaka, Japan) and digested in concentrated nitric acid (300 μL) at 95°C for 2 h. The volume of the digests was then made up to 10 mL by the addition of ultrapure water. The ^133^Cs concentrations of the samples were determined using inductively coupled plasma–mass spectrometry (ICP–MS; Agilent 7700x, Agilent Technologies, Tokyo, Japan) with 5 μg/L ^115^In as an internal standard. To analyze the mineral content of the candidate low-Cs-accumulating mutant lines and cross populations between *gmsos1* and ‘Enrei’, a sample of 10 seeds from each plant was dried at 105°C for 20–24 h; afterwards, the dried seeds were ground and digested as described above. The ^23^Na, ^39^K, and ^133^Cs concentrations of the seeds were also determined by ICP–MS using 5 μg/L ^115^In as an internal standard. The ^23^Na, ^39^K, and ^133^Cs concentrations of each sample were converted into the corresponding exchangeable element contents (mg/kg DW).

### Search for candidate SOS genes in soybean

SOS genes were searched in the published Williams 82 genome sequence and annotated genes of soybean (Wm82.a2.v1 at Phytozome v13.0, https://phytozome-next.jgi.doe.gov/info/Gmax_Wm82_a2_v1) using the amino acid sequences of the SOS genes in *Arabidopsis* (i.e., *AtSOS1* (*AT2G01980*), *AtSOS2* (*AT5G35410*), and *AtSOS3* (*AT5G24270*)) as queries. Genes were selected with a sequence homology of ≥60%; the genes that exhibited homology to the total length of amino acids were considered homolog candidates. The RNA-sequencing expression levels of the homologs in the soybean tissue, pod, root hair, leaves, root, nodules, seed, shoot apical meristem, stem, and flower were obtained from JBrowse of the Phytozome software (https://phytozome-next.jgi.doe.gov/).

### Screening of *gmsos1* mutants from the mutant library

The *gmsos1* mutants were isolated from the soybean mutant library, as previously described by Tsuda *et al.* (2015). To screen the candidate mutants, 1,536 M2ʹ soybean plants were subjected to high-resolution melting (HRM) analysis using an ABI ViiA7 Real-Time PCR System (Applied Biosystems, Pleasanton, CA, USA). The mutation-bearing DNA amplicons detected through HRM analysis were sequenced using the ABI PRISM 3500xL Genetic Analyzer (Applied Biosystems). The primers used for HRM and sequence analyses are listed in Supplemental Table 1. The primers used to confirm the sequences of the coding regions of *GmSOS1* (*Glyma.08G092000*) and of the *AtSOS2* and *AtSOS3* homologues (*Glyma.04G235900*, *Glyma.06G128700*, *Glyma.13G166100*, and *Glyma.17G113700*) in the *gmsos1* mutants are shown in Supplemental Table 2. The sequences of *GmSOS1* and of the *AtSOS2* and *AtSOS3* homologues in ‘Enrei’ were retrieved from DAIZU-net (https://daizu-net.dna.affrc.go.jp/ap/top).

The splicing pattern of *GmSOS1* in EnT-2953 was determined by performing reverse transcription–PCR (RT–PCR). Total RNA was isolated from the roots of EnT-2953 plants using the RNeasy Plant Mini Kit (Qiagen, Valencia, CA, USA); subsequently, portions of the total RNA (1 μg) were subjected to RT–PCR using the QuantiTect Reverse Transcription Kit (Qiagen) and the provided RT-primer mix (total volume = 20 μL). The resulting complementary DNA (cDNA) products (1 μL) were subjected to PCR analysis using two primer sets designed to amplify the cDNAs derived from *Glyma.08G092000* (5ʹ-ATGGAGGAAGAACAACAACAAC-3ʹ and 5ʹ-CTAGCGAAAAGATAGCGTGC-3ʹ). The amplified fragments were cloned into the pCR4 Blunt-TOPO vector (Invitrogen, Carlsbad, CA, USA), and the nucleotide sequences were confirmed using the 3500xL genetic analyzer (Applied Biosystems) and the BigDye-Terminator ver.3.1 cycle sequencing kit (Applied Biosystems). The primers are listed in Supplemental Table 3.

### Genotyping of *GmSOS1*

To genotype the young leaves obtained from the segregating populations of the *gmsos1* mutants (EnT-2953, EnT-3153, and EnT-3612), derived cleaved amplified polymorphic sequence (dCAPS) markers were developed to detect single-base substitutions that distinguish between the ‘Enrei’ and *gmsos1* mutants. The primers used for dCAPS marker analyses are listed in Supplemental Table 4. The amplicons were digested with *Bbs*I (EnT-2953), *Stu*I (EnT-3153), or *Hph*I (EnT-3612); subsequently, the restriction fragments were separated on a 3% agarose gel.

### Analysis of exchangeable ions in soil

After cultivation, soil samples from Fields A and B were collected at a depth of 0–15 cm from five locations (four corners and the center) and then mixed. The soil samples were dried at 40°C for 3 d and then sieved to <2 mm. After extraction for 2 h using a 1 M NH_4_OAc solution at a soil/solution ratio of 1:10, the exchangeable ^23^Na, ^39^K, and ^133^Cs concentrations (mg/kg DW) in the soil samples were determined by ICP–MS (Agilent Technologies) using 5 μg/L ^115^In as an internal standard.

### Analysis of ^137^Cs concentrations in plant and soil samples

The ^137^Cs concentrations of the seed and soil samples from Field A in 2018–2020 were determined using germanium detectors with multichannel analyzers (GC2520-7500SL and GCW2523–7905-30 U-ULB; Canberra Japan KK, Tokyo, Japan). The measurement results were decay-corrected to the harvest date (November 1) of the year of cultivation.

## Results

### Phenotypic screening of low-Cs-accumulating lines from a mutant soybean population

**Fig. 1.**
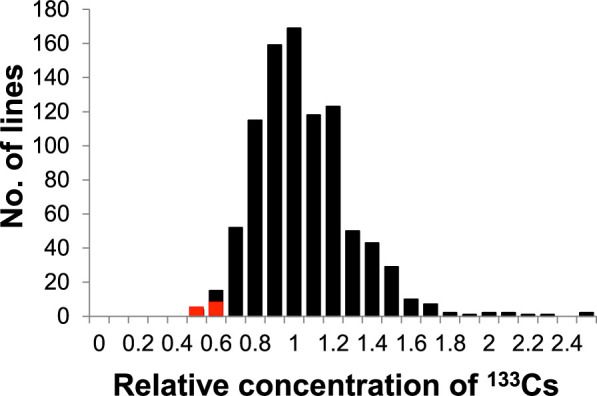
Frequency distribution of the seed ^133^Cs concentration in the soybean mutant population (905 lines). Seed ^133^Cs concentrations are shown relative to the average values of the population. The red blocks indicate the 10 lines selected as low-Cs candidates.

**Fig. 2.**
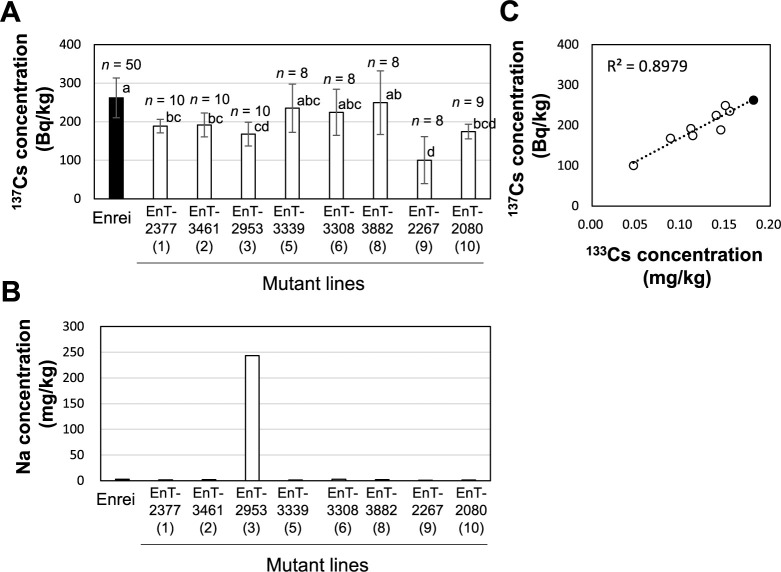
Seed Cs and Na concentrations in the low-Cs candidate mutant lines grown in Field A. The top 10 mutant lines with reduced seed ^133^Cs concentrations (Fig. 1) were grown in Field A (2018). The mutant lines and the original variety ‘Enrei’ were cultivated twice and ten times in five individuals, respectively. Two of the ten mutant lines were excluded from analysis because they did not grow normally and the number of harvested seed was small. Seed concentrations of (A) ^137^Cs and (B) Na. (C) Relationship between the seed concentrations of ^133^Cs and ^137^Cs. For the ^137^Cs concentration, the data shown in (A) are the means ± SD of the harvested individuals. For the Na and ^133^Cs concentrations, the same number of seeds from the harvested individuals was mixed in each replicate and subjected to ICP–MS analysis, and the average value of the two replicates is shown. The numbers in parentheses below the line names indicate the ranking of the seed ^133^Cs concentration, as shown in Fig. 1. Bars with the same letter of the same case do not differ significantly from one another (Tukey–Kramer multiple comparison test).

The M3ʹ mutant population used for screening the low-Cs-accumulating lines was harvested from a field in Tsukuba, Ibaraki, at a distance of >150 km away from the TEPCO–FDNPP. Given that the concentration of radiocesium (^134^Cs and ^137^Cs) in the seeds was too low to be measured, the nonradioactive Cs (^133^Cs) content was determined, which was the only natural isotope of Cs present in a measurable amount in the soybean seeds. Given that there is no convincing evidence of discrimination by biological systems among ^133^Cs, ^134^Cs, and ^137^Cs (Avery 1995), ^133^Cs is expected to behave exactly like radiocesium in soybean plants. Although the distribution pattern of the seed ^133^Cs concentration in the mutant population was continuous, four lines with a relative value of ≤0.5 were identified (Fig. 1). Considering the possibility of phenotypic errors due to the cultivation environment, the 10 mutant lines with the lowest seed ^133^Cs concentrations were selected and cultivated in Field A. K fertilizer was not applied in Field A; hence, the exchangeable K content of the soil was 65 mg/kg, which is significantly lower than the recommended value (>208 mg/kg) for suppressing Cs absorption (Table 1) (MAFF, NARO and NIAES 2015). The exchangeable ion concentrations of ^23^Na and ^133^Cs in Field A were 12.2 and 0.218 mg/kg DW, respectively (Table 1). Of the 10 mutant lines, two lines were excluded from the analysis because they did not grow normally and few seeds were harvested, thereby rendering it difficult to determine their exact phenotypes for Cs accumulation. Five mutant lines, namely EnT-2377, EnT-3461, EnT-2953, EnT-2267, and EnT-2080, exhibited significantly reduced seed radiocesium (^137^Cs) concentrations compared with the original variety ‘Enrei’ (Fig. 2A). Three mutant lines, namely EnT-3339, EnT-3308, and EnT-3882 showed no significant difference from ‘Enrei’. In addition, the ^133^Cs concentrations were also measured in these eight mutant lines and ‘Enrei’. It was found that the seed ^137^Cs concentrations of the mutant lines exhibited high positive correlations with their seed ^133^Cs concentrations, indicating that the use of ^133^Cs rather than ^137^Cs was acceptable for phenotypic screening (Fig. 2C). Among the homologous elements, the seed K concentration did not differ obviously between the mutant lines and ‘Enrei’ (Supplemental Fig. 1). However, the concentration of the homologous element Na was significantly higher in the EnT-2953 line compared to ‘Enrei’ (Fig. 2B), suggesting that the gene related to the sodium transport system may have undergone mutation.

### Identification of the *gmsos1* mutant lines

**Fig. 3.**
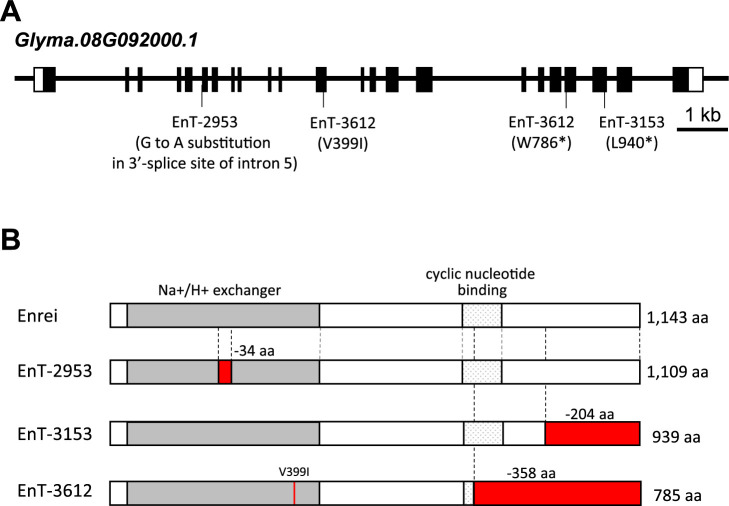
Gene structure of *Glyma.08G092000* as *GmSOS1*. (A) Schematic structure of *Glyma.08G092000*. Open and filled boxes indicate untranslated regions and exons, respectively. The mutation sites in EnT-2953, EnT-3153, and EnT-3612 are shown. (B) Structures of the *Glyma.08G092000* amino acids. The gray and dotted boxes indicate the Na^+^/H^+^ exchanger motif and the cyclic nucleotide binding motif, respectively. The red boxes and lines indicate deleted regions and amino acid substitutions in the mutant lines, respectively.

In rice, a mutant of *OsSOS2*, which encodes a serine/threonine-protein kinase required for the SOS pathway, has been reported to contain reduced radiocesium concentration in the grains (Ishikawa *et al.* 2017). Given that the *ossos2* mutant exhibited lower concentrations of Cs and higher concentrations of Na in the roots compared to the original variety, it was hypothesized that the EnT-2953 line may possess an aberrant SOS pathway involving SOS1, SOS2, and SOS3. A BLASTP search showed that soybean contains at least two homologs (*Glyma.13G166100* and *Glyma.17G113700*) of *AtSOS2* (*AT5G35410*) and two homologs (*Glyma.04G235900* and *Glyma.06G128700*) of *AtSOS3* (*AT5G24270*). However, only one homolog (i.e., *Glyma.08G092000*) was detected for *AtSOS1* (*AT2G01980*). This gene has previously been named as *GmSOS1* (Zhang *et al.* 2022), and it shares 64 and 61% amino acid sequence identities with *AtSOS1* and *OsSOS1* (*Os12g0641100*), respectively. RNA-sequencing expression data for nine soybean tissues, retrieved from the Phytozome database, revealed that *GmSOS1* is primarily expressed in the roots, suggesting that it may be involved in Na extraction in the roots (Supplemental Fig. 2). Considering that a mutated gene with one copy is more likely to influence the phenotype than a mutated gene with multiple copies, it was hypothesized that *GmSOS1* could be the causative gene for low Cs accumulation. The coding sequence of *GmSOS1* was subsequently confirmed using the genomic DNA of EnT-2953. A nucleotide G to A substitution at the 3ʹ-splite site of intron 5 was detected in the EnT-2953 line (Fig. 3A). Since EnT-2953 constitutes a homozygous mutant in the M2ʹ plants, it was considered a homozygous mutant in the M3ʹ generation used for phenotypic screening. The splicing pattern of EnT-2953 was confirmed using mRNA from the roots, and it was found that the sixth exon, which measures 102 bp and encodes 34 amino acids that form part of the Na^+^/H^+^ exchanger domain, was deleted in the transcripts (Fig. 3B).

Since the mutant population used in this study is thought to possess dense mutations throughout the entire genome (Tsuda *et al.* 2015), it is possible that genes other than *GmSOS1* are responsible for the reduced Cs concentration in EnT-2953; hence, additional *GmSOS1* mutant lines were searched in the same mutant library used for the phenotypic screening. Of the 1,536 mutant lines, two mutant lines (i.e., EnT-3153 and EnT-3612), which are likely to be functionally defective in *GmSOS1*, were identified. As shown in Fig. 3A, EnT-3153 and EnT-3612 carry a premature stop codon within the coding sequence of *Glyma.08G092000* (*GmSOS1*). In EnT-3153, a nucleotide substitution of T to A causes the replacement of leucine with a premature stop codon at amino acid position 940. In EnT-3612, a G to A nucleotide substitution causes the replacement of tryptophan with a premature stop codon at amino acid position 786. EnT-3612 also exhibited a G to A nucleotide substitution, resulting in the replacement of valine with isoleucine at amino acid position 399. Both mutant lines demonstrated a heterozygous mutant genotype in M2ʹ plants, and EnT-3153 was not included in the 905 lines subjected to phenotypic screening. It was confirmed that the three *gmsos1* mutants, namely EnT-2953, EnT-3153, and EnT-3612, possessed no mutations in the coding sequences of the *AtSOS2* (*Glyma.13G166100* and *Glyma.17G113700*) and *AtSOS3* (*Glyma.04G235900* and *Glyma.06G128700*) homologues.

### Seed ^137^Cs accumulation in the *gmsos1* mutants

**Fig. 4.**
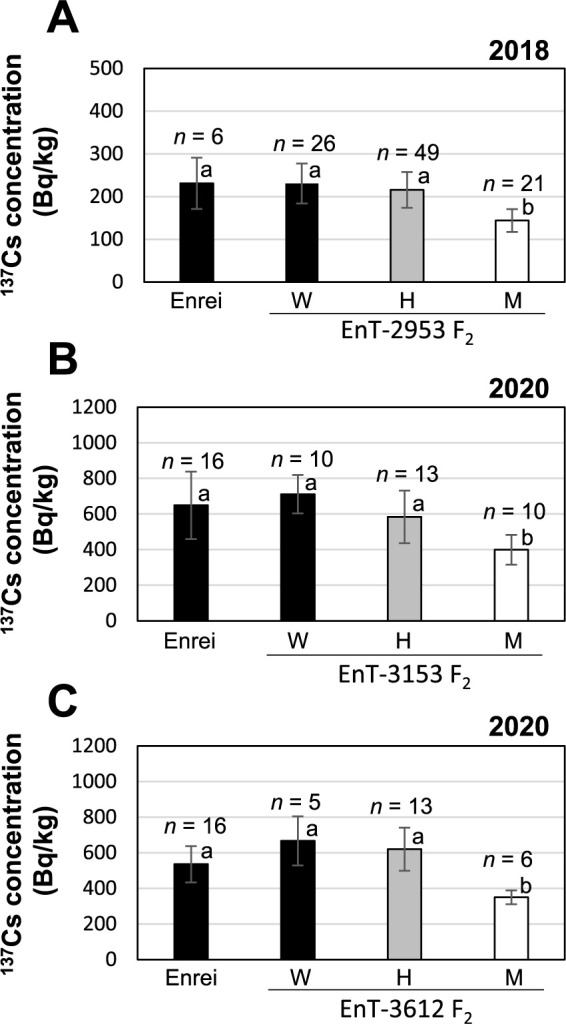
Seed ^137^Cs concentrations in three F_2_ populations between the *gmsos1* mutant and the ‘Enrei’ grown in Field A. (A) F_2_ populations between the EnT-2953 and ‘Enrei’ samples grown in 2018. (B) F_2_ populations between the EnT-3153 and ‘Enrei’ samples grown in 2020. (C) F_2_ populations between the EnT-3612 and ‘Enrei’ samples grown in 2020. The F_2_ plants were classified according to the *GmSOS1* genotype as follows: Wild type (W), heterozygous (H), mutant type (M). Data are reported as means ± SD. Bars with the same letter of the same case do not differ significantly from one another (Tukey–Kramer multiple comparison test).

**Fig. 5.**
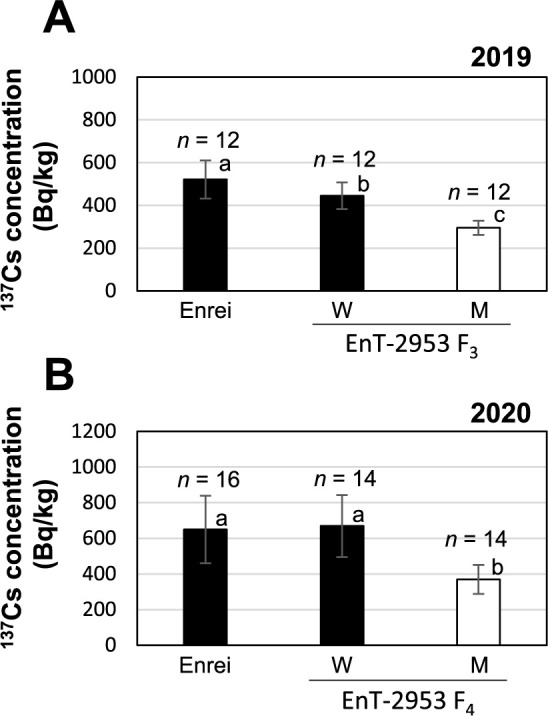
Seed ^137^Cs concentrations in the progeny lines from the F_2_ plants between the EnT-2953 and ‘Enrei’ samples in Field A. (A) F_3_ lines grown in 2019. (B) F_4_ lines grown in 2020. The F_3_ and F_4_ lines were bred through the self-fertilization of F_2_ individuals in which the *GmSOS1* genotype was confirmed. The *GmSOS1* genotypes were defined as follows: Wild type (W) and mutant type (M). Data are reported as means ± SD. Bars with the same letter of the same case do not differ significantly from one another (Tukey–Kramer multiple comparison test).

The exchangeable K content of Field A from 2018 to 2020 was 53–65 mg/kg (Table 1). In 2018, F_2_ plants derived from the cross between ‘Enrei’ and EnT-2953 were planted. After sowing, the genotype of each individual was confirmed using the dCAPS marker. The seed ^137^Cs concentrations of plants with the homozygous *gmsos1* mutant genotype were significantly lower than those of plants with the wild-type and heterozygous genotypes (Fig. 4A). In 2020, F_2_ plants derived from the crosses between ‘Enrei’ and either EnT-3153 or EnT-3612 were planted. Similarly, the seed ^137^Cs concentration in the F_2_ plants with homozygous mutant genotypes was significantly reduced (Fig. 4B, 4C). To evaluate the phenotypic stability for Cs accumulation in subsequent generations, the F_3_ and F_4_ progenies with homozygous mutant and wild-type genotypes, which were derived from crosses between ‘Enrei’ and EnT-2953, were planted in 2019 and 2020, respectively. The mutant genotype exhibited a significantly lower seed ^137^Cs concentration than the wild-type genotype in both generations (Fig. 5). These results suggest that the mutations that occurred in the EnT-2953, EnT-3153, and EnT-3612 lines are recessive; moreover, homozygous *gmsos1* mutant individuals possess lower Cs concentrations under low-K conditions.

### Agronomic traits in the *gmsos1* mutants grown in two field with different characteristics

**Fig. 6.**
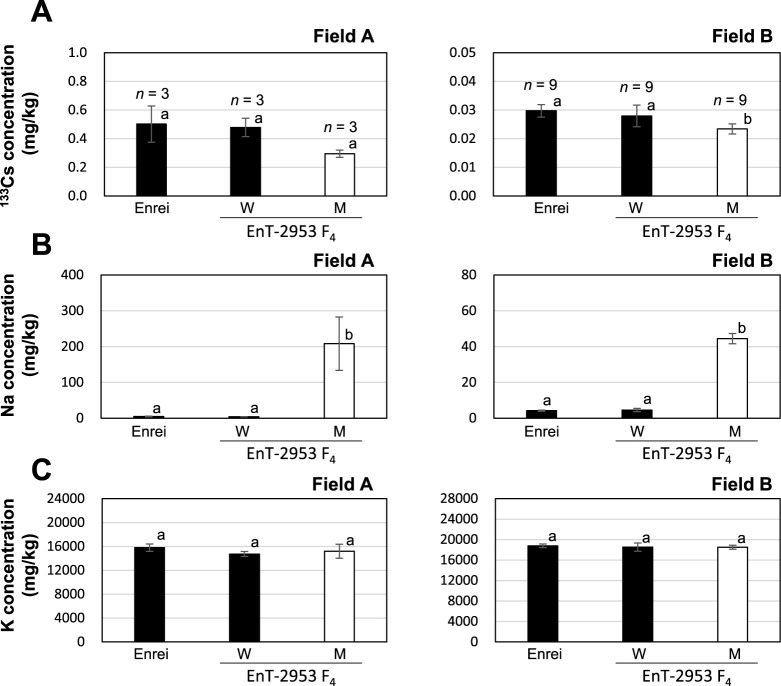
Seed concentrations of ^133^Cs, K, and Na in the F_4_ lines between the EnT-2953 and ‘Enrei’ samples in Fields A and B in 2020. (A) Seed ^133^Cs concentration. (B) Seed Na concentration. (C) Seed K concentration. The left and right sides show the data corresponding to Fields A and B, respectively. The F_4_ lines were bred through the self-fertilization of F_2_ individuals in which the *GmSOS1* genotype was confirmed. The *GmSOS1* genotypes were defined as follows: Wild type (W) and mutant type (M). Data are reported as means ± SD. Bars with the same letter of the same case do not differ significantly from one another (Tukey–Kramer multiple comparison test).

To investigate the phenotypic stability of Cs accumulation under different cultivation conditions, F_4_ lines of EnT-2953 were planted in Field B in addition to Field A. Since 100 kg/ha of K_2_O fertilizer was applied in Field B as a basal fertilizer, the exchangeable K concentration in Field B was 366 mg/kg, which is higher than the recommended value (>208 mg/kg) for suppressing Cs absorption (Table 1). The exchangeable concentrations of Cs and Na in Field B were lower than those in Field A. Owing to the high exchangeable K concentration and the low ^137^Cs concentration (<500 Bq/kg) in the soil from Field B, the seed ^137^Cs concentration was unmeasurable. Thus, ^133^Cs was measured instead of ^137^Cs, and the seed ^133^Cs concentrations were compared between Fields A and B. In Field B, the F_4_ plants with the mutant genotype demonstrated significantly reduced seed ^133^Cs concentrations than the ‘Enrei’ plants and the F_4_ plants with the wild-type genotype (Fig. 6A). In Field A, there was no significant difference in ^133^Cs concentrations between the mutant and wild-type lines at the 5% level. However, the mutant line showed lower ^133^Cs concentrations than the wild-type line. In addition, the mutant and wild-type lines significantly differed in terms of their ^137^Cs concentrations (Fig. 5B). Meanwhile, the mutant lines had a significantly higher seed Na concentration than the wild-type line in both fields (Fig. 6B), and the seed K concentration was not altered by the *GmSOS1* genotype in either field (Fig. 6C). A comparative cultivation test was performed in Fields A and B using the F_2_ population of EnT-3153, and similar results were obtained for the ^133^Cs, ^23^Na, and ^39^K concentrations (Supplemental Fig. 3). These results suggest that *gmsos1* reduces Cs accumulation and increases Na accumulation in soybeans regardless of the exchangeable K content and soil type.

**Fig. 7.**
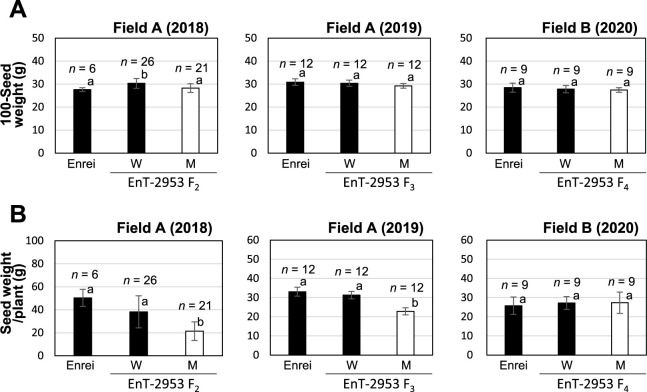
100-Seed weights and seed weights of the F_2_ plants between the EnT-2953 and ‘Enrei’ samples and its progeny lines grown in Fields A and B. (A) 100-Seed weight. (B) Seed weight per plant. The left, middle, and right regions show the data for the F_2_ plants in Field A, the F_3_ lines in Field A, and the F_4_ lines in Field B, respectively. The F_3_ and F_4_ lines were bred through the self-fertilization of F_2_ individuals in which the *GmSOS1* genotype was confirmed. The *GmSOS1* genotypes were defined as follows: Wild type (W) and mutant type (M). Data are reported as means ± SD. Bars with the same letter of the same case do not differ significantly from one another (Tukey–Kramer multiple comparison test).

To confirm the effect of *gmsos1* on agronomic traits, the 100-seed weight and seed weight per plant were also determined. In Field A, reliable seed weight data were not obtained in 2020 due to a lack of rainfall over an extended period, which resulted in a poor germination uniformity after sowing. Therefore, a preliminary comparison was performed in the EnT-2953 line using the data obtained from the F_2_ and F_3_ individuals cultivated in Field A, and the F_4_ individuals cultivated in Field B. No differences in the 100-seed weight were observed between the mutant and wild types (Enrei and wild-type F_2_, F_3_, or F_4_ individuals) (Fig. 7A). However, individuals with the *gmsos1* genotype cultivated in Field A exhibited a significantly lower seed weight per plant than the wild type, whereas no significant differences were observed in the seed weights between the mutant and wild types cultivated in Field B (Fig. 7B).

## Discussion

Soybean is a major crop grown worldwide. To ensure food safety, it is necessary to develop soybean varieties with low radiocesium accumulation and to elucidate the mechanism behind Cs accumulation in soybeans. In this study, candidate low-Cs-accumulating lines were searched in the mutant soybean population. Consequently, five low-Cs-accumulating lines were successfully identified based on phenotypic screening, and it was found that one of these lines was caused by a mutation in the *GmSOS1* gene, which encodes an Na^+^/H^+^ antiporter in the SOS pathway. To the best of our knowledge, there are no reports that *SOS1* is involved in plant Cs accumulation; however, it has been reported that the rice *SOS2* mutant (*ossos2*) contains reduced Cs concentrations in its grains and shoots (Ishikawa *et al.* 2017). Such a reduction indicates that the SOS pathway may also be involved in Cs accumulation in soybeans. In *ossos2* mutants of rice, Cs^+^ uptake by the roots was significantly decreased under low K^+^/Na^+^ conditions (Ishikawa *et al.* 2017). Because K^+^ transporters are suggested to mediate Cs^+^ uptake in plants (White and Broadley 2000, Zhu and Smolders 2000), the expression of four gene families (i.e., *OsHAK*, *OsAKT*, *OsHKT*, and *OsCNGC*) involved in K^+^ transport was considered. It was found that the transcript levels of several K^+^ and Na^+^ transporter genes, including *OsHAK1*, *OsHAK5*, *OsAKT1*, and *OsHKT2*, were significantly downregulated under low K^+^/Na^+^ conditions. This indicated that the reduced Cs^+^ uptake in *ossos2* mutants may be caused by the suppressed expression of these genes through K^+^/Na^+^ imbalance in the root, which in turn can be attributed to the *ossos2* mutation (Ishikawa *et al.* 2017). Based on homology searches of the soybean genome, soybean reportedly contains 70 putative K^+^ transporter genes (Rehman *et al.* 2017). However, very few studies have investigated the K^+^ transporters present in soybeans. Consequently, the mechanism responsible for reducing the seed Cs concentration in the *gmsos1* mutant is unknown at this time; nonetheless, it may be attributed to changes in the expression of K^+^ transporters, as in the case of *ossos2*.

In all field cultivation experiments involving siblings from the cross performed between the *gmsos1* mutants and ‘Enrei’, the individuals possessing the homozygous mutant genotype contained lower seed Cs concentrations than those with the homozygous wild-type genotype (Figs. 2A, 4, 5, 6A, Supplemental Fig. 3A). Although the degree of decline in the Cs concentration varied depending on the cultivation field and year, the Cs concentration was consistently lower than that of the wild type, ranging from 45.1 to 83.8%. Previously, Takagi *et al.* (2015) investigated a set of mini-core collections and identified several soybean germplasm accessions with low Cs accumulation. However, the position, number, and effect of genes controlling low Cs accumulation in these soybean germplasms are still unclear, which limits their potential use as breeding materials. In contrast, the low-Cs-accumulating mutants identified in our study originated from an elite soybean variety, which has favorable agronomic traits. Additionally, DNA markers associated with genes controlling low-Cs-accumulating mutants could be easily developed and applied in breeding practice. These factors make the mutants identified our study promising breeding materials for developing soybean varieties with low Cs accumulation.

In the current work, it was found that the *gmsos1* mutants contained higher seed Na concentrations than those with the homozygous wild-type genotype (Figs. 2B, 6B, Supplemental Fig. 3B). In contrast, the seed concentrations of K, an element homologous to Cs and Na, were similar between the mutant and wild-type plants (Fig. 6C, Supplemental Figs. 1, 3C). Notably, K is an essential element for plants, and since the seed K concentration in the *gmsos1* mutant was unaffected, it can be considered a useful breeding material. Furthermore, the *gmsos1* mutants showed no significant differences in their 100-seed weights compared to the wild type (Fig. 7A), and no obvious abnormalities were observed in seed germination or growth (data not shown). On the other hand, the mutants demonstrated significantly lower seed weights per plant than the wild-type plants under the low-K^+^ conditions of Field A (Fig. 7B). Although further cultivation tests are necessary to address this concern, it was considered that the yield may decrease in the mutants under K-deficient conditions because the seeds attempt to accumulate a certain amount of K despite the reduced amount of K uptake. In addition, considering that the loss-of-function *gmsos1* soybean created using the CRISPR-Cas9 system displayed an exceptionally increased salt sensitivity (Zhang *et al.* 2022), cultivation tests should be conducted in fields with high salt concentrations. Given that the soybean germplasm exhibits a diversity of salt tolerance phenotypes (Phang *et al.* 2008), the *gmsos1* mutation may reduce the salt tolerance, although its effect may vary depending on the genetic background. Several genes that play significant roles in soybean salt tolerance have been identified based on the quantitative traits loci (QTL) and genome-wide association studies using the soybean germplasm (Feng *et al.* 2023), and it may be possible to enhance the salt tolerance of the *gmsos1* mutant by utilizing these genes.

Based on the seed Cs concentration results obtained by phenotypic screening, four low-Cs-accumulating lines were identified in addition to the *gmsos1* mutant lines. More specifically, in addition to the *gmsos1* mutant EnT-2953, four lines stably showed significantly lower ^137^Cs concentrations than ‘Enrei’ (Fig. 2A). Among them, EnT-2267 had a lower ^137^Cs concentration than EnT-2953. In contrast to EnT-2953, this mutant line did not exhibit a high Na concentration (Fig. 2B), and so its causative gene was thought to differ from *GmSOS1*, potential rendering it an additional breeding material. In the future, it will be necessary to develop a segregating population by crossing with the original variety, evaluating the resulting phenotypic stability, and considering its usefulness as a breeding material. Moreover, it may be possible to identify additional mutants with low Cs accumulation levels using information obtained from other plant species. Previous analyses have shown that several genes, such as the high affinity K^+^ transporter (HAK) family and the cyclic nucleotide gated channels (CNGC), are involved in Cs accumulation in *Arabidopsis* and rice (Hampton *et al.* 2005, Qi *et al.* 2008, Rai *et al.* 2017). Mutants of the homologous of these genes may also lead to reduced Cs accumulation in soybeans. The mutant library used in this study has been applied not only to select mutants with phenotypes of interest through forward screening, but also to isolate specific gene mutants for functional analysis based on reverse genetics (Takagi *et al.* 2018, Yano *et al.* 2017, 2018). The soybean is of paleotetraploid origin, and as in the cases of *SOS2* and *SOS3*, multiple homologue candidate genes may also exist in *HAK* and *CNGC*. By accumulating mutations of each candidate gene through reverse genetics, it may be possible to obtain low cesium accumulation lines. Further identification of low-Cs mutants, such as EnT-2267, and their causative genes will be expected to lead to the development of ‘safer’ soybean varieties.

## Author Contribution Statement

K.T. and M.I. conceived and designed the research. K.T., Y.Y., Y.F., S.H., H.M., K.K., A.K., and T.A. conducted the experiments and prepared samples. K.T. and M.I. wrote the manuscript. All authors contributed to the development of this manuscript.

## Supplementary Material

Supplemental Figures

Supplemental Tables

## Figures and Tables

**Table 1.** Soybean cultivation fields used in this study

Name	Field type	Year	Sowing date	Fertilizer (kg/ha)				Exchangeable ion concentrations^1)^ (mg/kg)			^137^Cs concentration (kBq kg^–1^)
				N	P_2_O_5_	K_2_O		^23^Na	^39^K	^133^Cs	
A	Converted paddy field	2018	May 29	30	100	–		12.2	65	0.218	2.46
		2019	May 28	30	100	–		12.3	53	0.240	2.11
		2020	May 29	30	100	–		11.3	60	0.235	1.88
B	Upland field	2020	June 29	30	100	100		5.4	366	0.187	n.d.^2)^

^1)^ Exchangeable ion concentrations were measured using soil sampled after cultivation. ^2)^ ‘n.d.’ represents no data.
