# Supplementary material for: Identification of soybean mutants with low cesium accumulation, characterization of the causative gene, and field evaluation
Source: Breed Sci. 2025 Aug 9;75(4):281–91. doi: 10.1270/jsbbs.24069 (PMC13051629; doi:10.1270/jsbbs.24069)
Supplement: Supplementary file 1 — Supplemental Figures [file 75_281_s1.pdf]

doi: 10.1270/jsbbs.24069

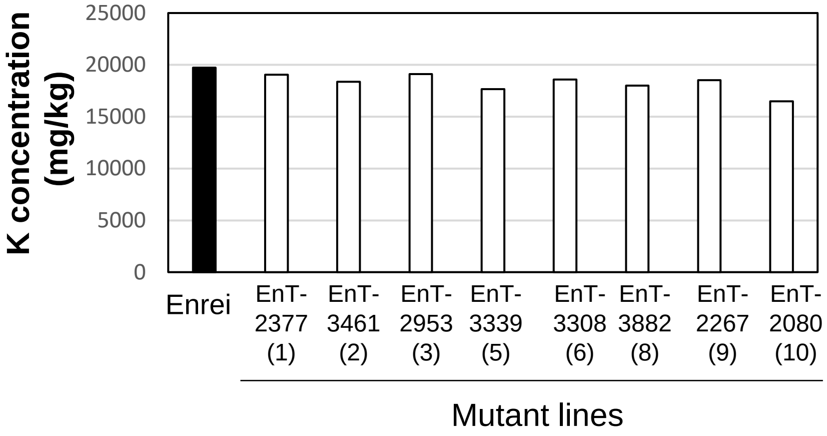

Supplemental Fig. 1. Seed K concentrations in low-Cs candidate mutant lines grown in Field A

The top 10 mutant lines with reduced seed ^{133}Cs concentrations (Fig. 1) were grown in Field A (2018). The mutant lines and original variety 'Enrei' were cultivated twice and ten times in five individuals, respectively. Two of the ten mutant lines were excluded from the analysis because they did not grow normally and the number of harvested seed was small. The same number of seeds from the harvested individuals was mixed in each replicate and subjected to ICP–MS analysis, and the average value obtained from the two replicates is shown. The numbers in parentheses below the line names indicate the ranking of the seed ^{133}Cs concentrations, as shown in Fig. 1.

Gene	Gene ID	Gene-level expression								
		pod	root_hairs	leaves	root	nodules	seed	sam	stem	flower
GmSOS1	Glyma.08G092000	5.389	9.553	2.245	22.412	11.58	9.668	7.667	5.319	6.476
GmSOS2	Glyma.17G113700	15.51	9.061	41.225	12.866	15.698	8.564	15.824	24.301	31.299
	Glyma.13G166100	10.165	5.243	10.712	10.671	12.308	5.427	3.865	7.486	12.174
GmSOS3	Glyma.06G128700	7.676	4.508	9.422	27.649	8.016	1.372	2.551	3.651	27.822
	Glyma.04G235900	1.413	6.078	7.251	26.716	8.096	3.441	3.013	5.388	3.019

sam: shoot apical meristem

Supplemental Fig. 2. RNA Seq expression of the candidate genes for *GmSOS1, GmSOS2* and *GmSOS3*

Gene expression data for the nine tissue were obtained form JBrowse for Wm82.a2.v1 at Phytozome v13.0 (https://phytozome-next.jgi.doe.gov/info/Gmax_Wm82_a2_v1).

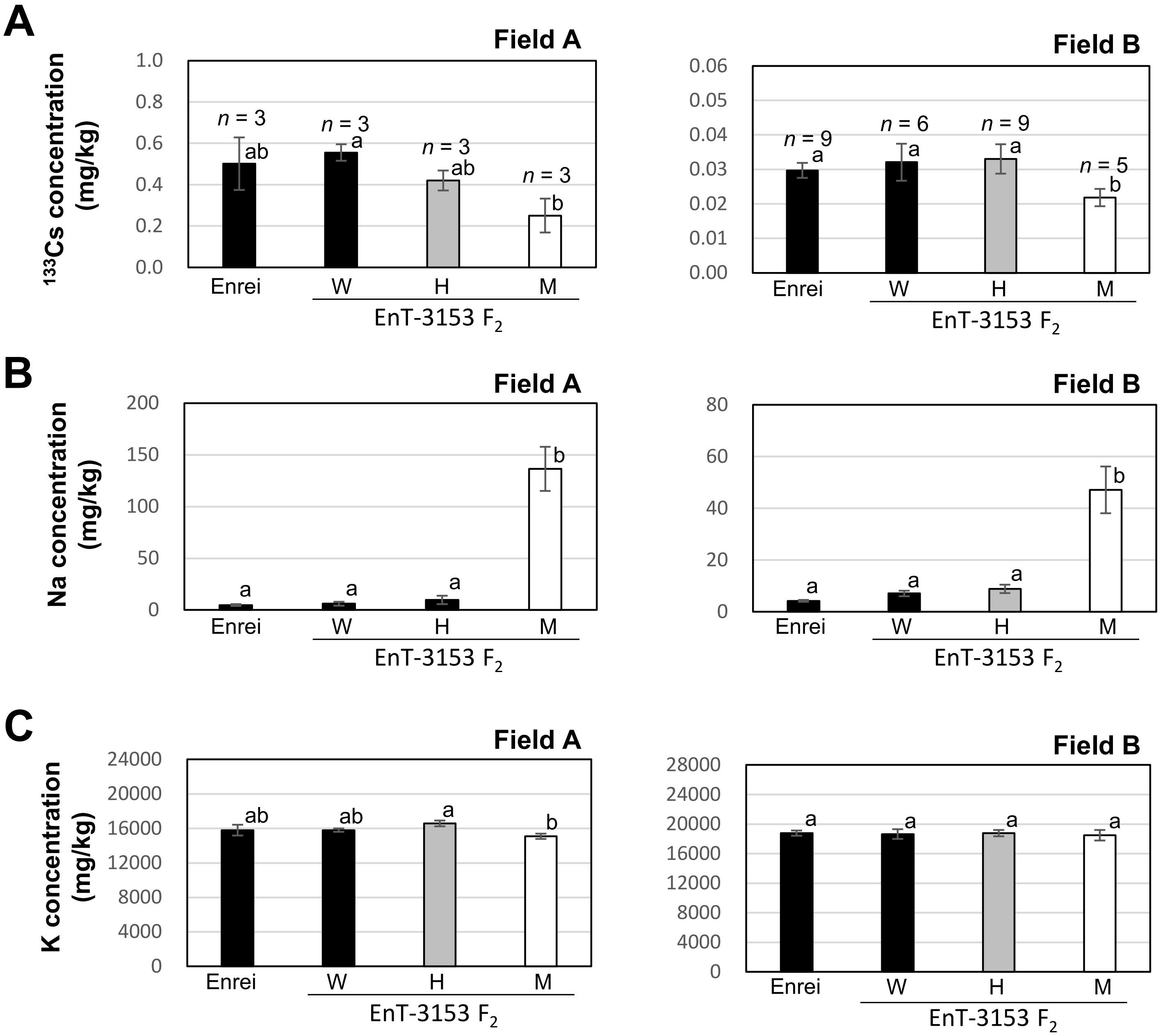

Supplemental Fig. 3. Seed concentrations of ^{133}Cs, K, and Na for the F_2 population between 'Enrei' and EnT-3153 in Fields A and B in 2020

(A) Seed ^{133}Cs concentration. (B) Seed Na concentration. (C) Seed K concentration. F_2 plants were classified according to the *GmSOS1* genotypes as follows: wild type (W), heterozygous (H), mutant type (M). Data are reported as means ± SD. Bars with the same letter of the same case do not differ significantly from one another (Tukey–Kramer multiple comparison test).
