# Supplementary material for: Identification of soybean mutants with low cesium accumulation, characterization of the causative gene, and field evaluation
Source: Breed Sci. 2025 Aug 9;75(4):281–91. doi: 10.1270/jsbbs.24069 (PMC13051629; doi:10.1270/jsbbs.24069)
Supplement: Supplementary file 2 — Supplemental Tables [file 75_281_s2.pdf]

doi: 10.1270/jsbbs.24069

**Supplemental Table 1** Primer sequences used in the HRM analysis

| Name | Amplified length (bp) | Forward primer (5'-3') | Reverse primer (5'-3') |
|---|---|---|---|
| 08G092000Ex1-1 | 158 | TTCCATCTCTGATTACTACTGCTG | ACTCTGGTCCCACGCAAG |
| 08G092000Ex1-2 | 159 | GCTCTGGGGATTGCTTGTAG | CCACAAGGCTAAGTGGTTCA |
| 08G092000Ex3 | 171 | GCTTTGGATGTGTTTCAACCT | TCAAATTGTACTCATAGAAGAATCCA |
| 08G092000Ex5 | 224 | CGATTATAGCTCATTTGTCACAATAC | TTCTTAGAACCTAGATTCATTTGG |
| 08G092000Ex6 | 167 | TGTTTCTGCATGTGATGGTTT | TTCACCACTAGTGCAACAAAGA |
| 08G092000Ex7 | 238 | TCATGTGACTGTTTGCTTGG | TCATCTTACCACAAGACATCCA |
| 08G092000Ex12 | 260 | CATGTTTGTGAAGTTTAAAAGAGTGA | ACAATGTCCGGGAAGAATTA |
| 08G092000Ex14 | 160 | CATTGCAACCCAGAAATCCT | CATAGAAGATCAGAAGAACCCTGA |
| 08G092000Ex15 | 262 | GCCCTTGTGTTTTGTTTACG | AAGTTACACAACTCTAAGGACTGAAA |
| 08G092000Ex16-1 | 145 | GCCTAATTTGAGAACTTATTGCT | TTCGGGAAATGGACGTTAGA |
| 08G092000Ex16-2 | 178 | TGAGCCTCTATGTGACTGGAAA | AGGCAAACTAATGAGATAGACTCA |
| 08G092000Ex17 | 295 | TTCAGAGCTTCCTCACATGG | CATCAGAGGTCACATTTAACTAGCA |
| 08G092000Ex18 | 224 | TTGTGCGACAATTTTCTGGT | TCAAACCATCAGATAACAGATAAACA |
| 08G092000Ex19-1 | 207 | TGTTTATCTGTTATCTGATGGTTTG | TTTGGCACCTTCCTTGTACAG |
| 08G092000Ex19-2 | 159 | TGCAAGTTGTACCAAGGAAATG | CATCTTGCTTTCCCACTGGA |
| 08G092000Ex20-1 | 136 | TGGAGTGGTGAAGGTATTAAATTCT | CCAGTCAACACTTCATAAATACCC |
| 08G092000Ex20-2 | 181 | TACATTTACGCATGGGAGCA | GAAGCCGTTAATGTCAAAGCA |
| 08G092000Ex21-1 | 161 | TCATTGGCCATGGTCTTACT | AGGGCAACTGAATGATGAGG |
| 08G092000Ex21-2 | 142 | CCATATTCATAAGAGGGGAAACA | TTACAGACAGCATATACGATCAGA |
| 08G092000Ex22-1 | 168 | GGCTTTGAAGCCTGATCATT | ATCCCCGGCATGTGATAAC |
| 08G092000Ex22-2 | 208 | CCGGGGATCATCCTCATAG | AGTAATCGATCAAATTTGGAATG |
| 08G092000Ex23-1 | 211 | GGCTTGCAGCAGAAATGTTA | TGTGCTTTGTGAAGGTGGAT |
| 08G092000Ex23-2 | 137 | TCCATGAGGTCACAAGACATG | CACAAAAGATTCTTCACCCTGA |

**Supplemental Table 2** Primer sequences used in sequence analysis of the coding region of *GmSOS1* and the homologues of *AtSOS2* and *AtSOS3*

| Name | Primer sequence (5'-3') |
|---|---|
| Gm08G092000-F1 | TGTTTTGTCGCGTACGACGTTACCA |
| Gm08G092000-R1 | ACCCAGCTATAGAAATCCACAAGGCT |
| Gm08G092000-F2 | GAAAACTATTACCTGCCAATCTATGTGACAG |
| Gm08G092000-F2-2 | GACAGCCCTTTTTTGCGTTTCCATACT |
| Gm08G092000-R2 | CATACCAAATTGAATGGGCCTGCACA |
| Gm08G092000-F3 | CAAGATCAGACATAGCTAGCATTTAACACTG |
| Gm08G092000-R3 | CTCTCATCTTACCACAAGACATCCAAGA |
| Gm08G092000-F4 | GATGTCTTGTGGTAAGATGAGAGATTATAACCT |
| Gm08G092000-R4 | GCAATGAACTCCCATTATTTTCATGTACTTGCA |
| Gm08G092000-F5 | CCCTCTAGTAACAAACCTTATTCAATGAGAG |
| Gm08G092000-R5 | GAAACAGGTGATGTAGAGATATTTTCATTTGGAATC |
| Gm08G092000-F6 | GTCAGGGTTCTTCTGATCTTCTATGTTC |
| Gm08G092000-F6-2 | TTACAGCAGGAGGAACCTCATCAG |
| Gm08G092000-F6-3 | AGGTTAAAGTAAAGGAAAGACACATCATCATAAGA |
| Gm08G092000-R6 | GAGAAGAAAGAATGGTGCTATGACTGAC |
| Gm08G092000-F7 | GGTTCAGAGCTTCCTCACATGG |
| Gm08G092000-F7-2 | TGCTAGTTAAATGTGACCTCTGATGTTTTC |
| Gm08G092000-R7 | AATCCCTGTTGAGCTGAAGGGACT |
| Gm08G092000-F8 | GCGTCTTTTAAGATATGCTGTTTATTGAGGAT |
| Gm08G092000-R8 | TGGATTCTCCTTTCAAAACCCTGCAAG |
| Gm08G092000-F9 | GTGTGGATTTACCTAGGCTCCATAAGA |
| Gm08G092000-R9 | CTTCAAGTAATAACACCCATTTCCAAATACGT |
| Gm13G166100-F1 | TCTTGGTTCTTTTCTCGTCACGCTAG |
| Gm13G166100-F1-2 | GTCAAGGTTCTGCTTCTAATCTTGCTTG |
| Gm13G166100-F1-3 | GGAATATATTAGAGTTTAATTCTTCTGAACACTGATAGTTCT |
| Gm13G166100-R1 | CTATTGATCTATTTCATGATTTTCATCACTAGTGATTTG |
| Gm13G166100-F2 | CAGCTCAGTTCATTAATTATACATTTCATGATTGAG |
| Gm13G166100-R2 | CAAGATGACTGCAAAAATAGATCATAACTAGTGA |
| Gm13G166100-F3 | CGATGTTATTCTCATTAAAAGATGTCACTAGT |
| Gm13G166100-F3-2 | GGAAAGTTCTTATACACTATTACAAAAGAAAAATTGCCA |
| Gm13G166100-R3 | CAAACAACAAAGATTGCGTTGATATCTGTGAC |
| Gm13G166100-F4 | CATGTAATCCTATTGTCACAGATATCAACGCA |
| Gm13G166100-F4-2 | CGCTTTTATTTTTTTCTCCTATGGTTCCCTTAAAAGT |
| Gm13G166100-R4 | CATTATGCTTTATACATTTCTGCCCGAACCA |
| Gm17G113700-F1 | TCAACTCTTTGCAATTGCGATCTTTCATTCCT |
| Gm17G113700-F1-2 | CAAGGTTCTGCTTCTAATCCTATGTGCT |
| Gm17G113700-F1-3 | GAATATGTTAGAGGTTAATTCTTCTGAACATTGATAATTCC |
| Gm17G113700-R1 | GACACTAAACTTAATACTATTGATCTATTTCATCACC |
| Gm17G113700-F2 | AGCTCGGTTCATCATTTATATATTTCATATGATTGAG |
| Gm17G113700-R2 | GGTGACTGCAATAATAGATCATAACTGACAG |
| Gm17G113700-F3 | TATAGATTTTGTCTACCATATGGTTCTGTCGG |
| Gm17G113700-F3-2 | GGAAAGTACATATATACTATTACAAAAACAAAATTGCCG |
| Gm17G113700-R3 | GCAAAAGTTACAAATAACAATGATCACATTGATATTTGATG |
| Gm17G113700-F4 | GTTACTCTGCATTATCTATTATGCCATATTATTCACT |
| Gm17G113700-F4-2 | CTACCAGTTCCCTTAAAGTAATTTGTCTTGTAG |
| Gm17G113700-R4 | TTGACAGCGTATGCTGTGCATTGTGA |
| Gm04G235900-F1 | TTCTAGTCAGCTCTATGCAATTTATCTGCCA |
| Gm04G235900-F1-2 | ACTAGTAGTATTTACTGCTGTAGCACATGATT |
| Gm04G235900-R1 | TGACATGACTGCCAATATTGTAGAACAAAACGA |
| Gm04G235900-F2 | CTATAGGCTAATAACTTACAAATTACAATACCCGA |
| Gm04G235900-F2-2 | GATTTCATGTTAATGTCTTCATTTCCCCTGAGT |
| Gm04G235900-F2-3 | GTCTTATCCATAATTCATAGATATATGTATGTGCTTGT |
| Gm04G235900-R2 | ACTATCAGCACAGGTTCAGCTACTAC |
| Gm06G128700-F1 | CAAGTTTTTGTCGGCTTTATACAATTTATATATGACTG |
| Gm06G128700-F1-2 | CATTCAGCACAAGCCAAGGCGT |
| Gm06G128700-R1 | CAGAGTCAAAAGTGATGAGACTGTCAATG |
| Gm06G128700-F2 | GTGCTATAGGCTAATTACTTATAGTATCCGAAGT |
| Gm06G128700-F2-2 | TGTAAGATCGACCCTTCCAGCC |
| Gm06G128700-F2-3 | GCTTAATTTTCTTTCATTTCCTTGATAAATTTACGTG |
| Gm06G128700-F2-4 | TGGCGTTCCTTAATTAAGTTGTGTACCTTC |
| Gm06G128700-R2 | GCTACTACAAACTACAAACTACAAAGAACTTCAGT |

**Supplemental Table 3** Primer sequences used in sequence analysis of the *GmSOS1* cDNA

| Name | Primer sequence (5'-3') |
|---|---|
| SOSFw1 | ATGGAGGAAGAACAACAACAAC |
| SOSFw2 | GATCCAGATCTTCTTTTAGCTG |
| SOSFw3 | CTCATTCTTAATGGAAGTTCACC |
| SOSFw4 | GAATGGGTCTTGCTTTTGGG |
| SOSFw5 | GGCACTTTCCTTATCAGTTAAG |
| SOSFw6 | AACAACAGCTAATATCCTAATGC |
| SOSFw7 | GAAATCCTCCTTTGGTTAAGC |
| SOSFw8 | GAACTCTTATTGCGGATTCCG |
| SOSFw9 | GCAAGGGCAATGCAGCTGAGC |
| SOSRv1 | CTAGCGAAAAGATAGCGTGC |
| SOSRv2 | CGATAGAAAAGAGTATAAACCAC |
| SOSRv3 | CCCAAGCCACAAAACAGATGC |
| SOSRv4 | GAAAGTGCCAAGGCAACTGCG |
| SOSRv5 | GAGAGCAGGAAGAAAAACAGC |

**Supplemental Table 4** Primer sequences used in dCAPS marker analysis

| Name | Amplified length (bp) | Primer sequence (5'-3') | Restriction enzyme |
|---|---|---|---|
| EnT-2953_dCAPS-F | 259 | AATGTATTTTATTTTTTGAAAAAAATTATTCTTCATTCTTGTCGA | *Bbs* I |
| EnT-2953_dCAPS-R | | ACAGATGCAATCCCAAAAGCAAGAC | |
| EnT-3153_dCAPS-F | 184 | GGGGAAACAATAGAAATTCCTCATCATTCAGT | *Stu* I |
| EnT-3153_dCAPS-R | | AAAAAAATATTACAGACAGCATATACGATCAGATACCTGAACAGGCC | |
| EnT-3612_dCAPS-F | 185 | AAAAGATGCATCTTTTGAAAATTTCTGTTTATCCGGTG | *Hph* I |
| EnT-3612_dCAPS-R | | GCTTCAAGAAAAATGCAGAATACCACGGAA | |

*Underling indicates mutated bases due to the addition of the restriction enzyme site
